# Neuroanatomical and Symptomatic Sex Differences in Individuals at Clinical High Risk for Psychosis

**DOI:** 10.3389/fpsyt.2017.00291

**Published:** 2017-12-22

**Authors:** Elisa Guma, Gabriel A. Devenyi, Ashok Malla, Jai Shah, M. Mallar Chakravarty, Marita Pruessner

**Affiliations:** ^1^Integrated Program in Neuroscience, Department of Psychiatry, Douglas Mental Health University Institute, McGill University, Verdun, QC, Canada; ^2^Department of Psychiatry, Cerebral Imaging Center, Douglas Mental Health University Institute, McGill University, Verdun, QC, Canada; ^3^Prevention and Early Intervention Program for Psychosis, Department of Psychiatry, Douglas Mental Health University Institute, McGill University, Verdun, QC, Canada; ^4^Department of Biological and Biomedical Engineering, Douglas Mental Health University Institute, McGill University, Verdun, QC, Canada; ^5^Department of Psychology, University of Konstanz, Konstanz, Germany

**Keywords:** structural MRI image analysis, sex differences, cortical thickness, clinical high risk for psychosis, brain morphometry

## Abstract

Sex differences have been widely observed in clinical presentation, functional outcome and neuroanatomy in individuals with a first-episode of psychosis, and chronic patients suffering from schizophrenia. However, little is known about sex differences in the high-risk stages for psychosis. The present study investigated sex differences in cortical and subcortical neuroanatomy in individuals at clinical high risk (CHR) for psychosis and healthy controls (CTL), and the relationship between anatomy and clinical symptoms in males at CHR. Magnetic resonance images were collected in 26 individuals at CHR (13 men) and 29 CTLs (15 men) to determine total and regional brain volumes and morphology, cortical thickness, and surface area (SA). Clinical symptoms were assessed with the brief psychiatric rating scale. Significant sex-by-diagnosis interactions were observed with opposite directions of effect in male and female CHR subjects relative to their same-sex controls in multiple cortical and subcortical areas. The right postcentral, left superior parietal, inferior parietal supramarginal, and angular gyri [<5% false discovery rate (FDR)] were thicker in male and thinner in female CHR subjects compared with their same-sex CTLs. The same pattern was observed in the right superior parietal gyrus SA at the regional and vertex level. Using a recently developed surface-based morphology pipeline, we observed sex-specific shape differences in the left hippocampus (<5% FDR) and amygdala (<10% FDR). Negative symptom burden was significantly higher in male compared with female CHR subjects (*p* = 0.04) and was positively associated with areal expansion of the left amygdala in males (<5% FDR). Some limitations of the study include the sample size, and data acquisition at 1.5 T. This study demonstrates neuroanatomical sex differences in CHR subjects, which may be associated with variations in symptomatology in men and women with psychotic symptoms.

## Introduction

Psychotic disorders, such as schizophrenia, show a great deal of variability in clinical presentation, disease course, and response to treatment. Some of the heterogeneity observed in patients suffering from psychosis may be related to sex-specific differences. Male patients typically present with an earlier age of onset, more severe negative symptom burden, and poorer functional outcomes (1). In contrast, females typically have higher affective symptom burden, but are more responsive to medications (2, 3).

There have been substantial observations suggesting that altered sex-specific adolescent neurodevelopmental and brain maturation trajectories play a role in the onset of mental illness (2, 4). Furthermore, magnetic resonance imaging (MRI) studies have reported sex differences in brain structure and function in patients suffering from schizophrenia, including larger ventricles, smaller temporal lobe, amygdala, and hippocampal volumes in male compared with female patients (5–7).

Since behavioral and neuroanatomical sex differences have been routinely observed in clinical studies and animal models of schizophrenia (8), it is important to better understand whether these differences (or a subset of them) are potentially observable in individuals considered to be at “high risk” for psychosis. Only a minority of individuals considered to be in a clinical high-risk (CHR) state actually develop psychosis; interestingly, neuroanatomical and clinical alterations similar to those observed in patients with psychosis have been observed in these individuals (9). These include gray matter (GM) volume reductions in the prefrontal, orbitofrontal, and limbic areas (10, 11). Although inconsistent, some studies have indicated that males at CHR have a greater risk of conversion to psychosis than females (12). Furthermore, males at CHR have more severe negative symptoms and lower functioning at entry to service, whereas women often present with more severe affective symptoms (12), similar to clinical findings observed in frank psychosis. A recent study demonstrated smaller left hippocampal volume in male compared with female CHR subjects, and an association between reduced hippocampal volume and blunted cortisol awakening response (13). Similar results have also been observed in first-episode psychosis (FEP) (14).

Building on the findings of Pruessner et al. in FEP (14) and CHR (13), the goal of this study was to perform a detailed neuroanatomical characterization of sex differences in CHR individuals in the cortex and limbic structures using sophisticated volumetric, cortical thickness (CT), and surface-based mapping techniques. We also aimed to characterize sex-specific symptom profiles, and how these map onto neuroanatomical sex differences. Given findings of a recent meta-analysis on MRI studies in CHR subjects, we predicted reduced hippocampal and amygdala volumes in individuals at CHR (15). Since cortical thinning in the frontal and temporal lobes has been well replicated in FEP and chronic schizophrenia (16), we predicted that we would observe thinning in these areas in CHR individuals. Pruessner and colleagues used the same data that were analyzed in this paper to investigate relationships between CHR neuroanatomy and stress reactivity (13). Following up on this work, we hypothesized that these structural alterations be more pronounced in male than female patients. We further predicted higher negative symptom load in males to be associated with brain anatomy in limbic structures (hippocampus and amygdala) given their involvement in emotional processing (17). A better understanding of sex-specific morphological alterations in individuals at CHR may help in developing novel biomarkers that are not typically associated with neuropsychiatric disorders and high-risk populations. Given the sex differences in clinical presentation in individuals at CHR, it would be important to determine if there are also brain morphometric differences. We believe that with our tools, we may be able to detect more subtle sex-specific differences in development and disease pathophysiology.

## Materials and Methods

### Subjects

Twenty-six antipsychotic naive individuals identified as being at CHR for psychosis (13 men, mean age 20.2 ± 3.1, 13 women, mean age 21.2 ± 3.5) were recruited from the Clinic for Assessment of Youth at Risk (CAYR) at the Prevention and Early Intervention Program for Psychosis at the Douglas Hospital Mental Health University Institute, Montreal, Canada (18, 19). CHR status was established using the Comprehensive Assessment of at Risk Mental States instrument (20). Twenty-nine healthy control subjects (15 men, mean age 22.3 ± 3.6, 14 women, mean age 23.8 ± 4.2) were recruited from the community through newspaper advertisements. The absence of any history of mental illness, drug abuse, or current medication was documented with the Structured Clinical Interview for DSM-IV Axis I Disorders, non-patient edition (21). The study was approved by the McGill Institutional Review Board. All participants gave informed consent for study participation.

### Symptom Assessment

The severity of psychotic symptoms was assessed by a trained professional at the CAYR clinic with the brief psychiatric rating scale (BPRS) for CHR subjects (not CTL) (22) in the CHR group. Separate BPRS ratings for positive, negative, depressive, and manic symptoms were determined based on results from a factor analysis (23). Overall functioning was assessed with the global assessment of functioning scale (24). Clinical follow-up period after intake was 2 years. Only four CHR subjects (two males) developed a psychosis during the follow-up period. Five CHR (four females) subjects were treated with antidepressant medication at the time of the MRI scan.

### Image Acquisition

Magnetic resonance images (MRI) were acquired on a 1.5 T Siemens Magnetom Vision scanner at the Montreal Neurological Institute within 6 months of the baseline assessment at the CAYR clinic (between 2006 and 2008). One cubic millimeter isotropic images were obtained using a T1 weighted, standard three-dimensional gradient-echo pulse sequence, with a field of view of 256 mm, repetition time of 22 ms, echo time of 9.2 ms, and flip angle of 30°.

### Image Pre-Processing and Total Brain Volume (TBV) Extraction

T1-weighted MR images were converted to the MINC file format^1^ and processed using the minc-bpipe-library pre-processing pipeline from the CoBrA Laboratory tools.^2^ Pre-processing consisted of a two-step whole-scan bias field correction, with and without an approximate brain mask using N4ITK (25). Image extents were cropped to remove excess data around the head, improving subsequent image processing steps. A brain mask was computed using the BEaST patch-based segmentation technique (26) to calculate TBV and for use in subsequent processing steps (see below).

### CT Analysis

CT and surface area (SA) were estimated using the CIVET pipeline (version 1.1.12; Montreal Neurological Institute at McGill University, Montreal, QC, Canada). In this pipeline, the pre-processed T1-weighted images (see Image Pre-Processing and Total Brain Volume (TBV) Extraction) were registered to MNI-space *via* the MNI ICBM 152 model (27). Next, voxel-wise tissue classification was performed to parcellate tissue into GM, white matter (WM), and cerebral spinal fluid (28, 29). Deformable models were then used to create a WM and GM surfaces for each hemisphere separately, resulting in four surfaces of 40,962 vertices each (30, 31). GM to WM surface distances were determined using the *t*-link metric (32). Thickness estimates were then blurred using a 20-mm surface-based diffusion kernel and non-linearly aligned to a template (32). SA was measured at the mid-cortical surface (33). All cortical models were aligned through an automated surface-based registration algorithm (34). The automated anatomical labeling (AAL) atlas was used to label the resampled surfaces generated from the CIVET pipeline to compute average thickness and total SA in 92 cortical areas. The AAL template was originally defined on the Colin27 brain MNI atlas (35), and registered to the ICBM surface model to enable use with surface-based models (36). Quality control was performed by EG to determine whether the cortical surfaces generated respect the anatomy of the cortex.

### Automatic Segmentation of Hippocampus, Amygdala, Basal Ganglia, and Thalamus

#### Volume of Hippocampus and Amygdala

Bilateral hippocampus and amygdala volumes were extracted from T1 images using the automatic multi-atlas segmentation algorithm, MAGeT Brain (37, 38). To generate hippocampus and amygdala volumes, we used five high-resolution atlases onto which the structures had been expertly segmented (39). These 5 atlases were used to label a set of 21 template scans, chosen to be representative of the variability in the data. Labeling was achieved through standard model-based segmentation procedures using the ANTs algorithm for atlas-to-template non-linear registration (40). All other subjects were then warped to the 21 templates, yielding 105 possible candidate segmentations. Final segmentations were decided using a voxel voting procedure (41). This configuration has been shown to be as or more accurate than other information-based label fusion methods in the literature (42). Following segmentation, quality control was performed by EG; visual inspection was used to determine whether the segmented labels generated through MAGeT brain respect the anatomical borders of the regions. The basal ganglia and thalamus segmentations were performed using the same algorithm but using a different input atlas (43), as described in Section 1.1 in Data Sheet S1 in Supplementary Material.

#### Vertex-Wise SA

Surface-based analyses of the hippocampus and amygdala were performed as described previously (44, 45). They were estimated with a marching cubes algorithm (46) and corrected using the AMIRA software package (Visage Imaging, San Diego, CA, USA), and then transformed back into the native space of each subject. The 21 transformations mapping each subject to the templates and to the final atlas (an average representation of the 5 atlases) were concatenated and averaged across the template library to increase accuracy and precision (47). This generated 105 surfaces per subject, which were then merged *via* a vertex-by-vertex median vote procedure for each structure for each subject. SA at each vertex was estimated to be the average of all adjoining triangles in the mesh. Finally, SA values were blurred (5 mm) prior to statistical analyses. Similar methods were applied to the basal ganglia structures (see Section 1.2 in Data Sheet S1 in Supplementary Material).

#### Surface Displacement

Surface displacement was also assessed for the hippocampus and amygdala as previously described (48) and was used as a metric for measuring shape. Surface displacement is estimated using the dot product between the average non-linear deformation vector derived from the average atlas-to-subject transformation, and the surface normal to provide a local measure of inward (concavity) or outward (convexity) of displacement along the normal. The basal ganglia surface displacement was processed in the same way. This provides a meaningful and complementary metric to vertex-wise SA (49).

### Statistical Analysis

We first tested for sex-by-group interactions covarying for age on TBV measures using a general linear model using the RMINC software package^3^. Next, we analyzed hippocampus and amygdala volumes as a ratio of TBV to account for variations in overall brain size, using the same model described above (sex × group interaction with age covariate). Multiple comparisons corrections were not applied to these statistical tests as we had *a priori* hypotheses regarding the fact that these structures would be affects both by sex and diagnosis group. For exploratory analysis of basal ganglia measures, see Section 1.3 in Data Sheet S1 in Supplementary Material.

A general linear model was used to test for sex-by-group interactions with age as a covariate on average CT and total SA measures from the regions generated from the AAL atlas segmented. False Discovery Rate (FDR) was used for multiple comparisons correction (50, 51). The same model was applied vertex-wise on CT and cortical SA measures, and to hippocampal and amygdala SA and displacement measures.

BPRS symptom scores were tested for sex differences using the Wilcoxon rank sum test, as the data were not normally distributed. We first assessed sex differences in negative symptom presentation. Because negative symptom load was significantly higher in males, and there was lack of variance in scores of female CHR participants (11 of 13 female CHR participants had a score of 3), we examined the relationship between negative symptom severity and brain anatomy in males only. We performed a linear model to test for relationships between BPRS negative symptom score and the hippocampus and amygdala for CHR subjects, covarying for age. Hippocampus and amygdala volume, displacement, and SA measures were tested.

### Power Analysis

A power analyses were conducted *post hoc* to assess the statistical power of our sample in relation to the observed means and variances. CHR and CTL were compared within each sex for a representative set of areas (CT) and vertices (displacement measures); the required sample size was plotted to obtain power = 0.80 (α = 0.05) using the observed means and a range of variances. CT areas chosen for the power analysis included a representative region from our significant results (right postcentral gyrus), as well areas where differences between CHR and CTL groups would be expected based on previous meta-analyses (right superior temporal gyrus and left parahippocampal gyrus) (51). For displacement measures, the power analysis was performed using mean displacement measures for the vertex of peak displacement, as well as a vertex where there was less statistical significance for displacement differences (weak vertex). *A priori* power analyses were not conducted as these data were used retrospectively. We chose to perform a *post hoc* power analysis calculation similar to Schaer and colleagues (52) to determine whether the group differences we observed through our statistical analyses were sufficiently powered and how many subjects would be required to sufficiently power a study of this kind. We believe that sex differences in CHR is an important topic and that providing this information would be critical to future groups performing studies centered around this research question.

## Results

### Socio-Demographic and Clinical Factors

Table S1 in Supplementary Material provides details on demographic characteristics of male and female CHR and control participants. The CHR group was found to be significantly younger than the control group (*t* = 1.17, *p* = 0.04), with no significant age difference between males and females in either subgroup (all *p* > 0.05). CHR subjects were also less likely to have completed a high-school level education compared with controls (χ^2^ = 6.83, *p* = 0.009). No other socio-demographic differences were present between CHR and controls, male and female subjects overall, nor when they were split into CHR and control subgroups, including ethnicity, relationship status, tobacco smoking, and cannabis use (all *p* > 0.05).

### Volumetric Findings

**Figure 1:**
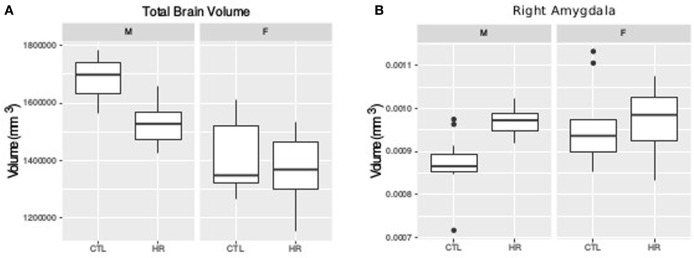
Sex difference in total brain volume (TBV) and left amygdala volume measures. **(A)** Sex-by-diagnosis interaction on TBV displayed in boxplots where the mid-line represents the median of the data; the box shows the first and third quartiles, and the vertical line represents the range of the data (*p* = 0.08). **(B)** Sex-by-diagnosis interaction for right amygdala volume displayed in boxplots (*p* = 0.08).

For TBV a sex-by-diagnosis interaction did not reach significance (*t* = 1.76, *p* = 0.08), however CHR males had smaller TBV than healthy control males, whereas there was no difference between CHR and control females (Figure 1A). Significant main effect of group (*t* = −3.67, *p* = 0.0008) and sex (*t* = −5.83, *p* < 0.0001) suggest that TBV is smaller in CHR than CTL, and smaller in females than males. For the right amygdala, we observed larger differences in proportional volume relative to TBV between CHR males and control males than between female CHR subjects and female controls, however, this interaction did not reach significance (*t* = −1.80, *p* = 0.08) (Figure 1B). In addition, there was a significant main effect of group for both the left and right amygdala, with larger volumes in the high-risk group (left: *t* = 3.39, *p* = 0.002; right: *t* = 2.60, *p* = 0.01). The main effect of sex was also significant bilaterally, with larger proportional volume in females (left: *t* = 2.87, *p* = 0.007; right: *t* = 2.61, *p* = 0.014). There were no significant sex-by-group interactions, or main effects for hippocampal volume between CHR and healthy controls (*p* > 0.05), apart from trend level sex differences for proportional volume (females > males, right: *t* = 1.78, *p* = 0.08).

In exploratory analyses on the basal ganglia and thalamus, we found bilaterally larger striatal volume in CHR individuals (uncorrected *p* = 0.026 right and *p* = 0.020 left; see Section 2.1 in Data Sheet S1 in Supplementary Material), but did not observe any significant sex-by-group interactions. For a summary of mean volumes (±SD) and statistics, see Tables S1 and S2 in Supplementary Material, respectively.

### Sex Differences Observed in the Cerebral Cortex

**Figure 2:**
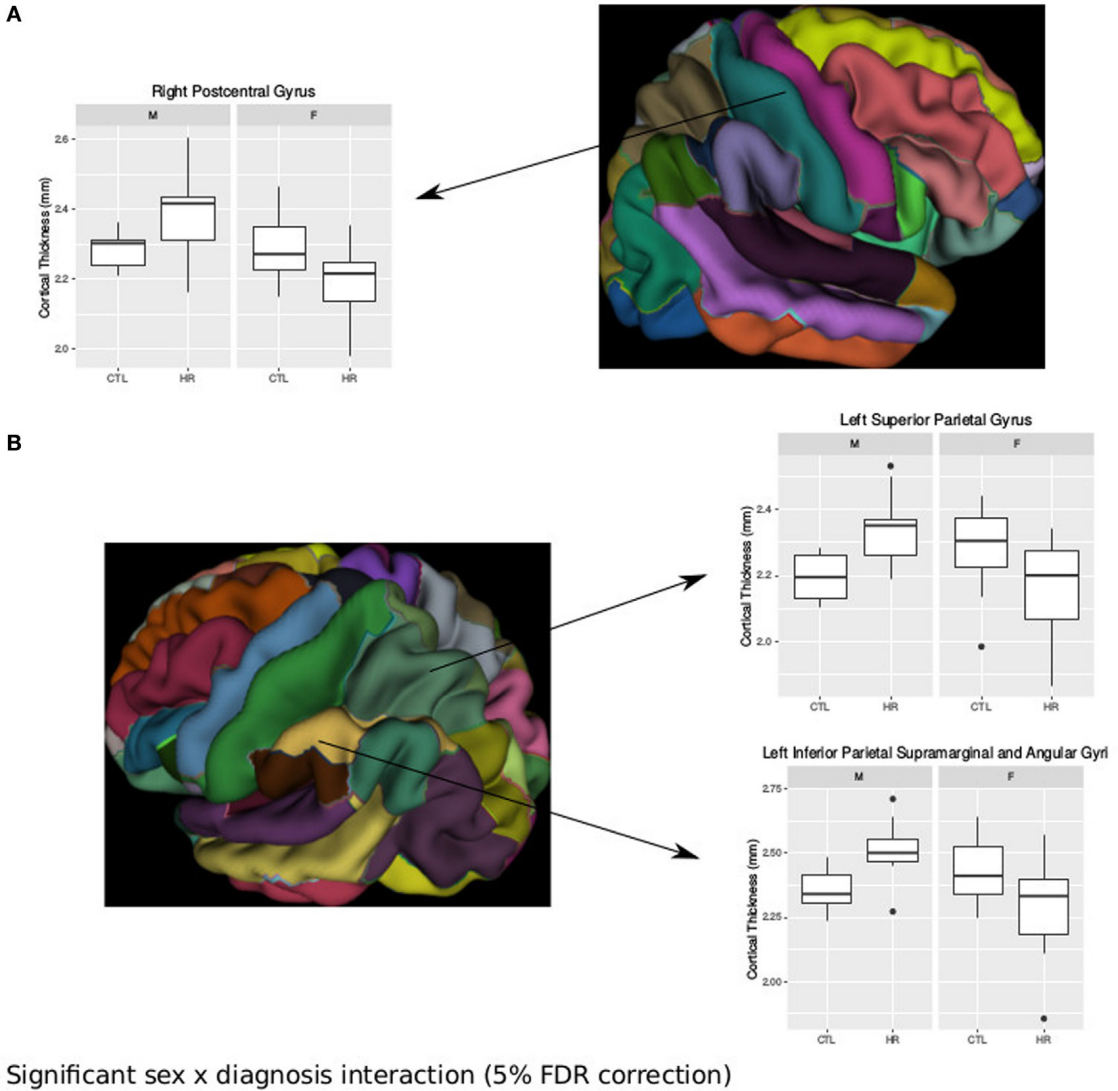
Sex differences in cortical thickness (CT) in individuals at clinical high risk and healthy controls. **(A)** Significant sex-by-diagnosis interaction in the right postcentral gyrus with boxplot of average thickness of the right postcentral gyrus [<5% false discovery rate (FDR)], and representation of automated anatomical labeling (AAL) cortical parcelation of right hemisphere. **(B)** Significant sex-by-diagnosis interaction in CT of the left hemisphere. Representation of the AAL cortical parcelation of left hemisphere, with boxplot to illustrate significant interaction for average thickness of the left superior parietal gyrus, and left inferior parietal supramarginal and angular gyri (<5% FDR).

**Figure 3:**
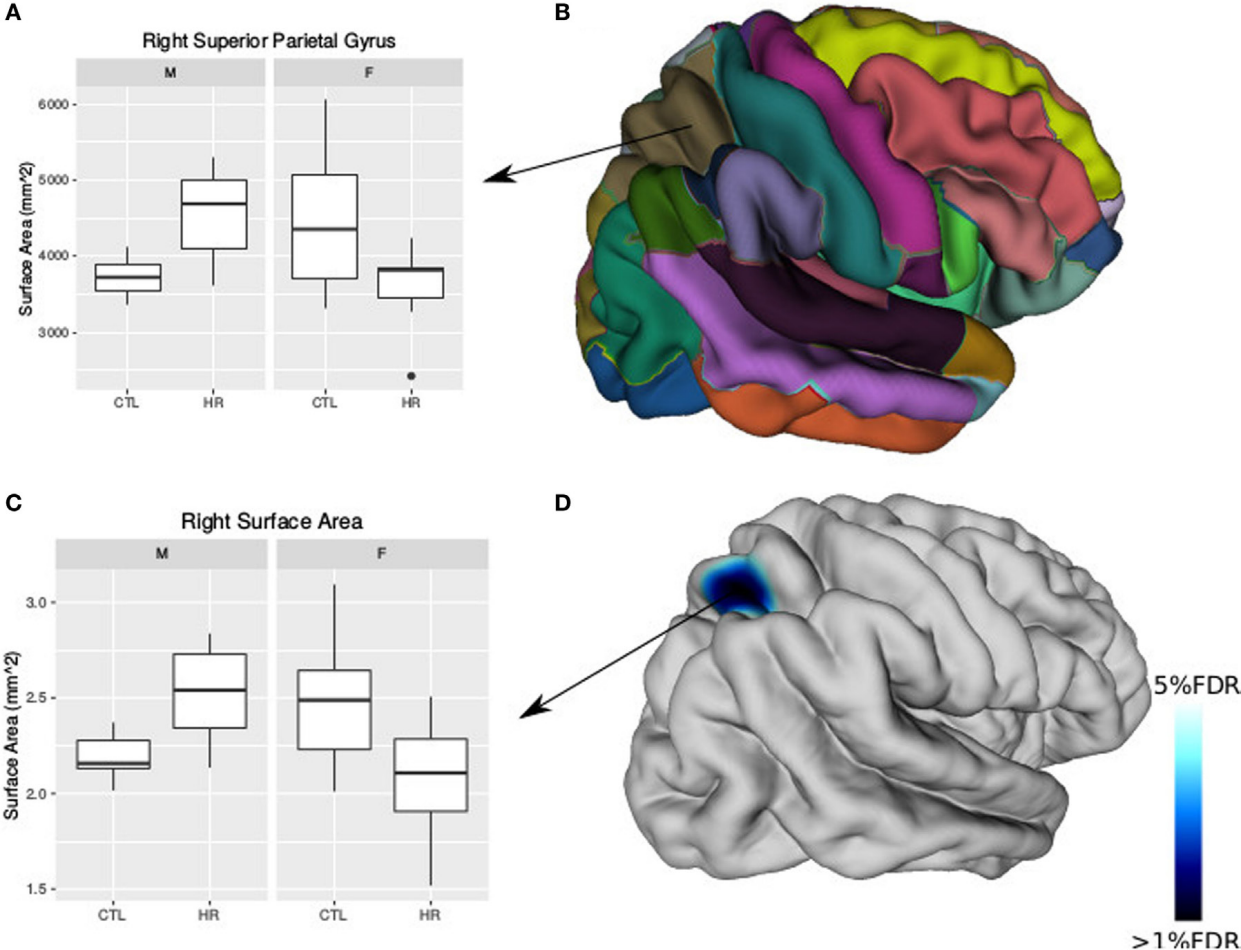
Sex differences in surface area (SA) in individuals at clinical high risk and healthy controls. **(A)** Boxplot representing significant sex-by-diagnosis interaction in the right superior parietal gyrus SA. **(B)** Automated anatomical labeling cortical parcelation of right hemisphere. **(C)** Boxplot of peak vertex representing significant sex-by-diagnosis interaction on cortical SA [<5% false discovery rate (FDR)]. **(D)** Cortical representation of significant sex-by-diagnosis interaction for SA with blue column representing *t*-values above the 5% FDR threshold mapped onto the average cortical surface.

Using the AAL atlas parcelation of the cortex, we found statistically significant (<5% FDR) sex-by-group interactions in mean CT of the right postcentral gyrus [mean thickness (millimeters): male CTL = 2.27, male CHR = 2.35, female CTL = 2.27, female CHR = 2.20], the left superior parietal gyrus (mean thickness: male CTL = 2.19, male CHR = 2.34, female CTL = 2.27, female CHR = 2.16), and the left inferior parietal supramarginal and angular gyri (mean thickness: male CTL = 2.53, male CHR = 2.51, female CTL = 2.43, female CHR = 2.29) (Figure 2). The direction of the interaction was consistent in these areas; males at CHR showed a thicker cortex than control males, whereas females at CHR had a thinner cortex relative to control females. Subthreshold (i.e., do not survive FDR correction) sex-by-group interactions were noted in vertex-wise CT measures in similar regions (*p* < 0.05, uncorrected) (Figure S1 in Supplementary Material). We also observed a significant sex-by-group interaction in the SA of the right superior parietal gyrus [total SA (square millimeter): male CTL = 29,793, male CHR = 45,795, female CTL = 35,463, female CHR = 39,565] (<5% FDR). This result was also significant in the vertex-wise SA measures in the same region (<5% FDR) (Figure 3).

### Sex Differences in Displacement Metrics

**Figure 4:**
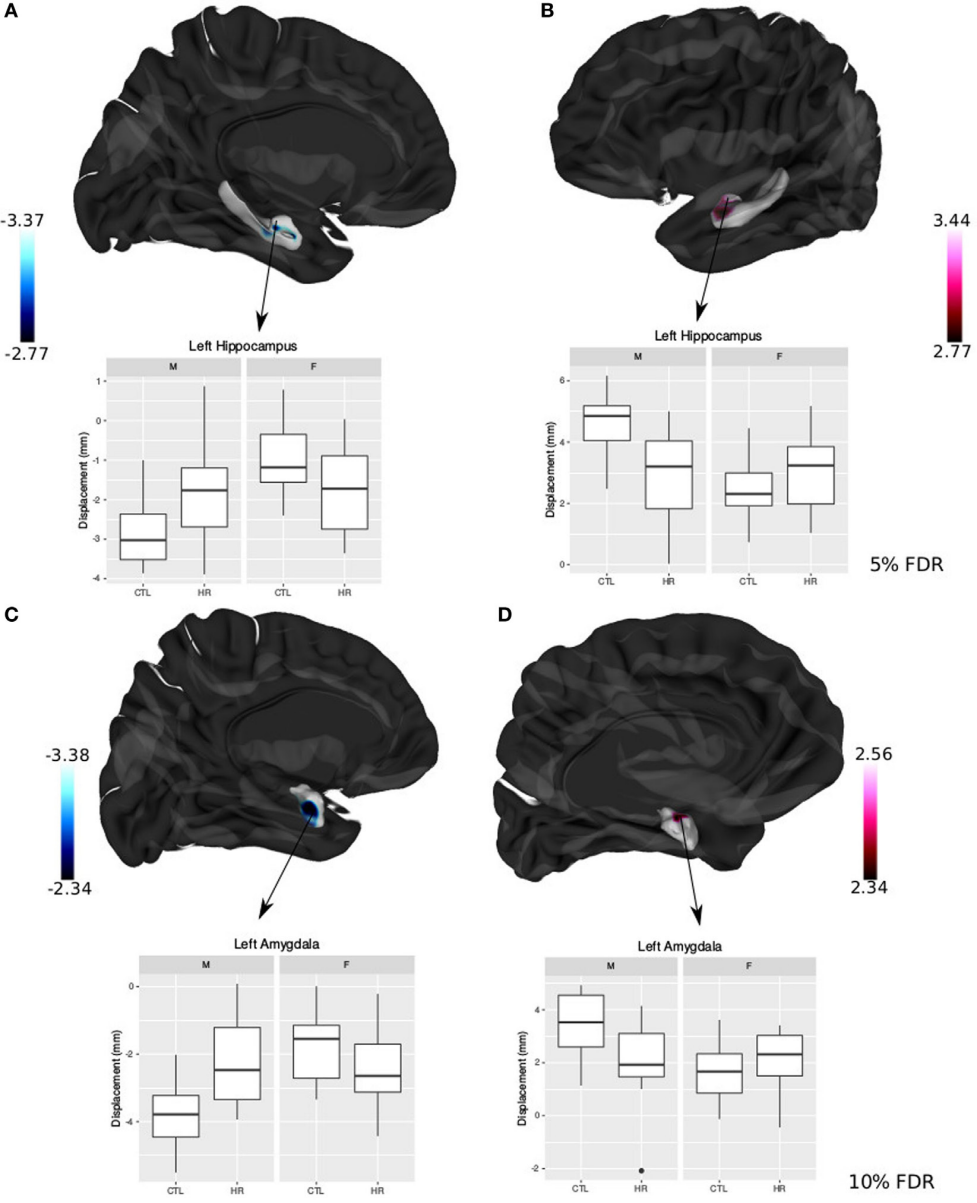
Sex differences in hippocampal shape and amygdala shape. **(A,B)** Significant sex-by-diagnosis interaction for left hippocampus displacement measures; *t*-statistics overlaid on group average hippocampus surface [<5% false discovery rate (FDR)]. **(C,D)** Significant sex-by-diagnosis interaction for left amygdala displacement measure in the lateral amygdala; *t*-statistics overlaid on group average amygdala surface (<10% FDR). In blue, there is inward displacement of females at clinical high risk (CHR) relative to CTL females, and outward displacement of CHR males relative to CTL males. In pink, the opposite is found, with inward displacement of CHR males relative to CLT males, and outward displacement of CHR females relative to CTL. All boxplots represent displacement values of peak voxel. Color bar denotes *t*-statistics with more significant values in darker shades.

Significant sex-by-group interaction was observed in the displacement measures across the left hippocampus and amygdala. In the left hippocampus, we observed outward displacement ventrally and inward displacement dorsally in males at CHR compared with control males (<5% FDR). Conversely, in females we observed inward displacement ventrally, and outward displacement dorsally (Figure 4). In the left lateral amygdala, males at CHR showed outward displacement posteriorly compared with control males. In contrast, CHR females showed inward displacement posteriorly compared with control females (<10% FDR). In the medial amygdala, inward displacement was observed in CHR males relative to controls, whereas outward displacement was present in females at CHR relative to controls (Figure 4).

### Sex Differences in Symptom Severity and Neuroanatomy

**Figure 5:**
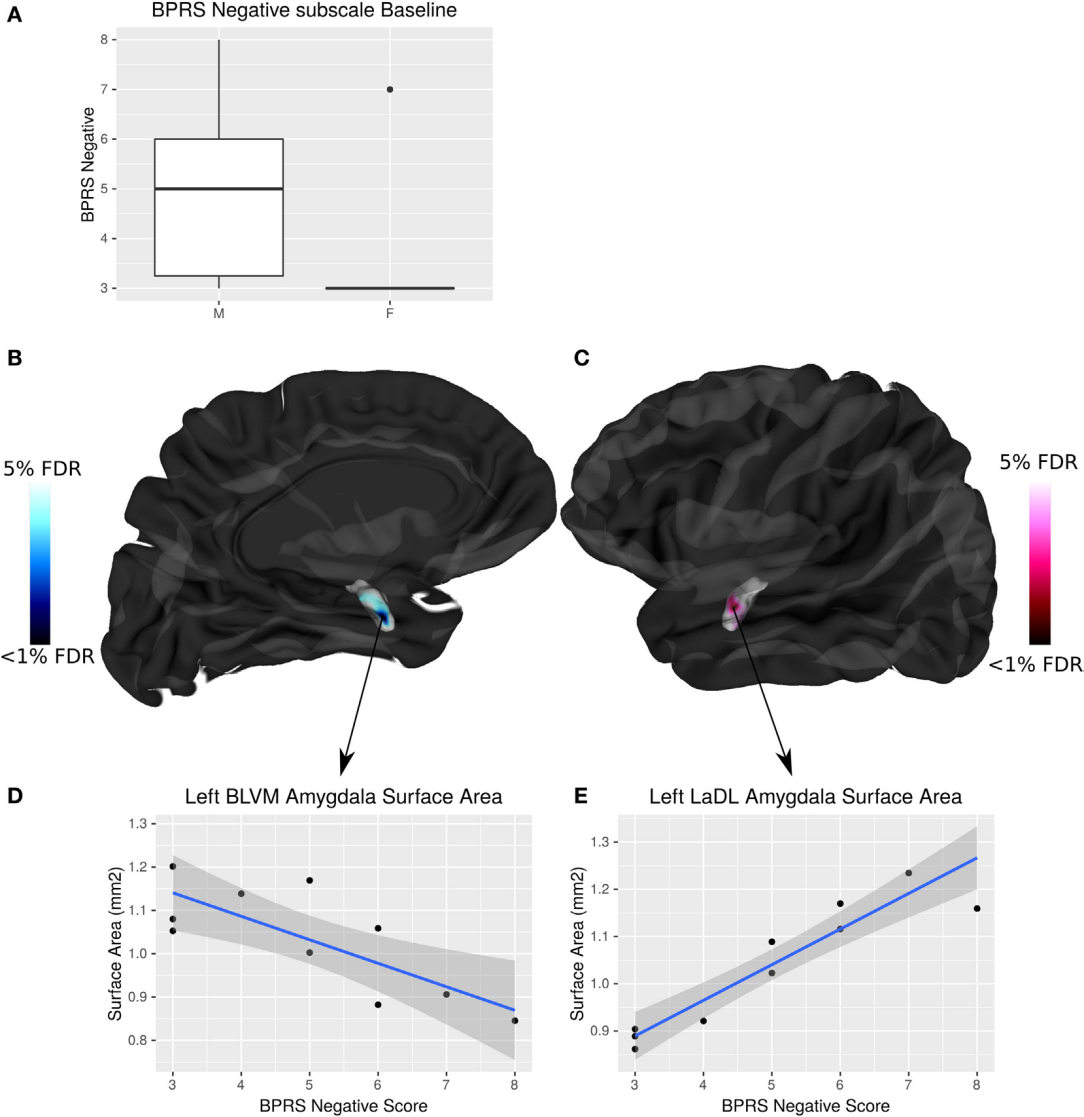
Sex differences in brief psychiatric rating scale (BPRS) negative symptom load and its interaction with amygdala surface area (SA) in males at clinical high risk (CHR). **(A)** Boxplot showing that males have significantly higher BPRS negative symptom load than females (*p* = 0.04). **(B,C)** Highlight areas of the amygdala SA (corresponding to the baso-lateral ventromedial nucleus in blue, and lateral nucleus in pink) associated with negative symptom severity in males at CHR. *t*-Statistics thresholded between 20 and 5% false discovery rate (FDR). **(D)** Linear regression showing negative relationship between SA and symptom severity. **(E)** Linear regression showing positive relationship between SA and symptom severity.

Negative symptom burden was higher in CHR males than females (*W* = 19, *p* = 0.04). A significant relationship between negative symptom severity and left amygdala SA was found in male CHR subjects. As negative symptom load increases, SA of the baso-lateral ventromedial amygdala decreases and the lateral amygdala SA increases in males at CHR (<5% FDR) (Figure 5). There were no associations between symptomatology and hippocampal volumes or shape that survived FDR correction.

### Power Analyses

#### Average CT

**Figure 6:**
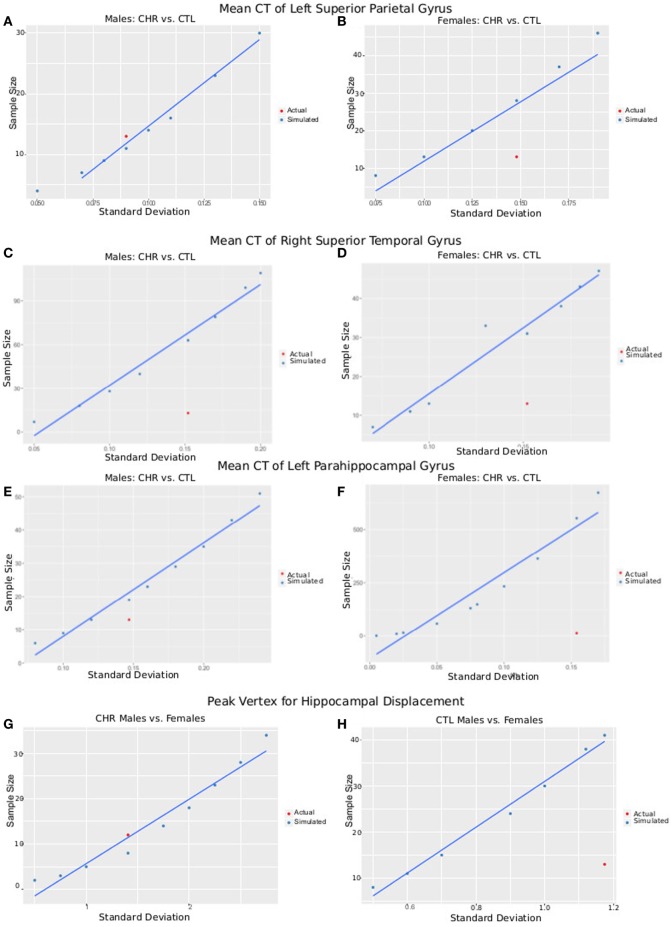

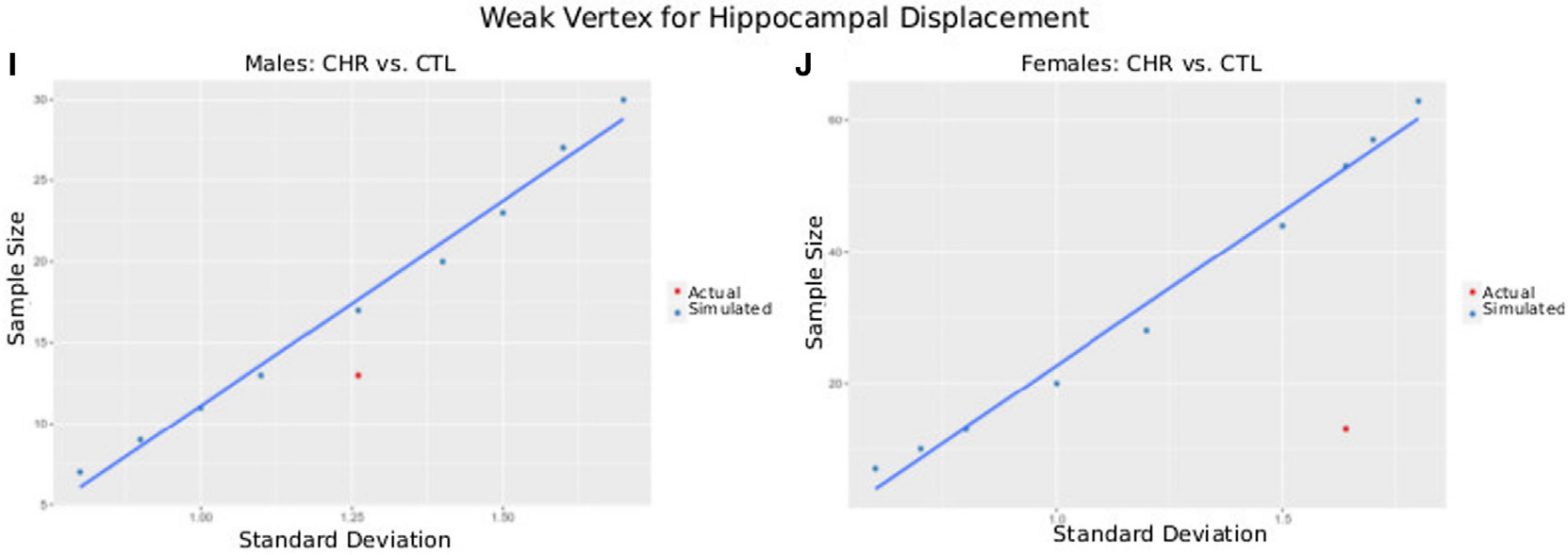
Plots of SD versus required sample size for power = 0.8 and α = 0.05. Using either the peak vertex or mean cortical thickness (CT) values, we calculate the sample size that would be required to obtain 80% power given α = 0.05 and a variety of SDs. These are plotted in blue as simulated data. In red, we plot the actual SD for each result versus the sample size of each group [clinical high-risk (CHR) male = 13, CTL male = 15, CHR female = 13, CTL female = 14], allowing a comparison of our sample size versus the sample size required to measure the effect. **(A,B)** Plots show data from CT of the left superior parietal gyrus of male subjects **(A)**, where CHR males had thicker cortex than CTL males, and female subjects **(B)**, where CHR females had thinner cortex than CTL females. **(C,D)** Plots show data from CT of the right superior temporal gyrus (a region in which we did not observe group by sex interactions) of male subjects **(C)** and female subjects **(D)** comparing CTL with CHR. **(E,F)** Plots show data from CT of the left parahippocampal gyrus (a region in which we did not observe group by sex interactions) of male subjects **(E)** and female subjects **(F)** comparing CTL with CHR. **(G,H)** Plots show data from the peak vertex for left hippocampal displacement for male subjects **(G)**, and female subjects **(H)**, where significant sex-by-group interactions were observed. **(I,J)** Plots show data from a weak vertex (i.e., where the group × sex interaction was weaker) for left hippocampal displacement for male subjects **(G)**, and female subjects **(H)**, where significant sex-by-group interactions were observed. Overall **(A–I)** give confidence that the sample size of our study is sufficient to observe the reported effects in male subjects, but that we may be slightly underpowered for the females. Further, we may have been underpowered to detect group differences in other regions of interest **(C–F)**.

The postcentral gyrus was selected as a representative cortical region where sex-by-group interactions were observed. The mean CT of the four groups and a range of variances were used to simulate required sample size for a study with power = 0.8, and α = 0.05. Simulated sample sizes are plotted against the actual sample size and variance in Figure 6; the plot shows that our sample size was sufficient to detect differences between CHR (*n* = 13) and CTL (*n* = 15) in the superior parietal gyrus for males, but not females (CHR, *n* = 13; CTL, *n* = 14). Our sample of females would have required 18 females per group. For the right superior temporal gyrus, we were underpowered for both the males and females; samples of 63 and 31 would have been necessary, respectively. For the left parahippocampal gyrus, we were also slightly underpowered for males in which a sample size of 19 would have been required; we were very underpowered for the female group in which a sample of 553 would have been required.

#### Vertex-Wise Displacement

The same procedure was performed for hippocampal displacement measure. For the peak voxel, we had sufficient power to detect differences between CHR and CTL in males but not females, for whom 41 females would have been necessary. For the weak voxel, we were slightly underpowered to detect differences in the male sample, as we would have required 16 subjects (3 more than what we had in our CHR group, and 1 more than what we had in our CTL group). For the females, again we were underpowered, as a sample of 46 would have been necessary (similar to what we observed at the peak voxel).

## Discussion

This is one of few studies to examine sex differences in the neuroanatomy and symptomatology of individuals at CHR for psychosis. As predicted, we observed that males compared with females at CHR have more dramatic anatomical alterations relative to their same-sex controls. Specifically, we found that male CHR individuals show decreased TBV but increased CT in the parietal lobe, whereas female CHR subjects show no overall differences in TBV but have a thinner parietal cortex compared with CTLs. Different surface displacement in the amygdala and hippocampus in male and female CHR subjects suggests a sex-specific morphological patterning of these structures in the CHR group. In addition, the observed higher negative symptom severity in males at CHR was associated with increased areal expansion of the left amygdala SA.

Areas in which we detected neuroanatomical alterations are consistent with other studies performed in patients with schizophrenia, FEP, or at high risk for psychosis (53, 54). However, there is limited information as to whether these parietal lobe alterations are sex dependent. In normative development, it has been shown that, relative to cerebrum size, females have more GM volume in their parietal lobe than males (55–57). While we observed a similar pattern in our control males and females (i.e., females had thicker cortex in the parietal lobe gyri, including the postcentral, superior partial, and inferior parietal supramarginal and angular gyri), females at CHR had a thinner cortex whereas males at CHR had a thicker cortex compared with their same-sex controls. Surprisingly, we did no detect any differences in the frontal or temporal lobes. Although only a small subset of individuals at CHR actually convert to FEP, this cortical pattern is similar to what has been observed in FEP and schizophrenia, with a more feminized neuroanatomy in affected males, and more masculinized neuroanatomy in affected females (58, 59).

In individuals at CHR findings on bulk hippocampus volumes have been somewhat inconsistent (60). In the absence of total volume changes, more localized reductions have been reported in the body and tail of the hippocampus in individuals at CHR (61). Similarly, findings on amygdala volumes are heterogeneous, as some report volume decreases, whereas others report no change (60, 62). To the best of our knowledge, shape analysis of the amygdala has not been performed in individuals at CHR.

Hippocampal and amygdala alterations have seldom been investigated in the context of sex differences. This is troubling, as these regions are known to be different in healthy males and females, with larger hippocampus observed in females and larger amygdala observed in males when accounting for TBV (59, 63). Therefore, understanding how neuroanatomical sex differences present in the context of mental illness could provide insight into the neurobiology of the illness. A previous study from our group investigating the hippocampus in FEP observed bilaterally reduced hippocampal volume in males but not females (14). Reports on amygdala volumes are more heterogeneous; both amygdala volume increases and decreases have been reported in females with schizophrenia, with either no changes or decreases in male amygdala volume (58, 64).

While we did not find any significant sex-by-group differences in bulk hippocampal volume, we did observe significant surface displacement in the left anterior hippocampus with opposite directionality in males and female CHR relative to same-sex controls. In our previous study in this CHR group, using different statistical and segmentation approaches, we had found smaller left hippocampal volume in CHR males compared females (13). Thus, using different methodologies, both studies have identified abnormalities in the left hippocampus and point to striking sex differences in brain anatomy between male and female CHR subjects. We only observed trend level volume differences (normalized to TBV), with an increase of volume in males at CHR, but not in females. Surface-based investigation of the anatomy revealed more subtle sex-specific differences in displacement in both lateral and medial areas in the left amygdala. We observed both inward and outward displacement, which could explain why no overall volume changes were observed in the hippocampus, and why only trend level differences were present for amygdala volumes.

We did not observe any sex-specific differences in our exploratory analysis of the thalamus and basal ganglia. Thalamic and striatal volume alterations have often been observed in psychosis (65, 66). However, these areas have not been found to differ in a sex-dependent manner in previous work, or in this study.

Males in our CHR group suffer from more severe negative symptoms, which is in line with findings from the psychosis literature (67). The association we observed with negative symptom load and amygdala SA is interesting, as the amygdala is implicated in emotional and cognitive processing (68). Few studies have examined associations between anatomy and symptom severity in CHR individuals. Bernasconi and colleagues previously reported a negative correlation between hippocampal volume and negative symptom load in individuals at high risk for psychosis (69). Associations between the amygdala and negative symptom load have not been reported in CHR individuals, but have been observed in more severe psychotic disorders such as schizophrenia. Gur and colleagues found a positive correlation between amygdala volume and negative symptom load in males with schizophrenia, with an opposite relationship in women (58). Although we did not find a correlation between symptom severity and hippocampal and amygdala volume, we observed a relationship with the SA of these structures in males at CHR. It is possible that we did not detect bulk volume changes because the level of neuroanatomical abnormality and symptom severity in the CHR group is subtle.

Brain morphology is inherently multi-dimensional. CT and SA are governed quite clearly by different genetic and environmental processes. SA architecture is governed by genetic factors and is thought to be “hardwired” during gestation and development while CT is more sensitive to environmental exposures (70). This is important to investigate in the context of CHR, as both genetic and environmental factors may be contributing to their symptoms and neuroanatomical alterations. This is also true for subcortical morphology (as measured by displacement), as it is more sensitive to detecting subtle group differences. In fact, previous work has shown that brain shape alterations may be more sensitive to the underlying processes of brain development and disease progression instead of volume (44, 45, 48).

### Limitations

The results of this study should also be considered in light of its limitations. Our sample size is modest as these individuals are difficult to recruit. We were sufficiently powered to detect differences between CHR and CTL males, but not for the females. This may explain why some of our results do not reach the threshold for significance following multiple comparisons corrections, and why males seem to have more severe deficits. Additionally, we were slightly underpowered in areas where we would have expected differences, such as the superior temporal lobes, so it is possible that with a larger sample, we would have been able to detect subtler group differences. Furthermore, our scans were collected at 1.5 T, which often results in decreased contrast and longer acquisition times than at 3 T, although it should be noted that our segmentation tools have previously been validated at 1.5 T (45, 48, 71, 72). We believe our methods are sensitive to subtle changes in our regions of interest; however, it is possible that we do not capture certain differences that could have been identified by performing voxel-based morphometry over the whole brain. There has been recent interest in determining how to correct for sex-specific brain volume in the context of neurodevelopmental disorders. While there are many methods that can be used, some have suggested determining differences in the context of structure-to-brain allometry may be critical for accurately capturing group differences (73). These types of considerations should be addressed in future work. Furthermore, the conversion rates observed in our study population (4%) are lower than those typically reported and may be a limitation; although the nature of CHR populations is fairly heterogeneous, mean (95% confidence interval) conversion rates, based on approximately 2,500 CHR individuals is estimated to be of 18% (12–25%) at 6 months of follow-up, 22% (17–28%) at 1 year, 29% (23–36%) at 2 years, 32% (24–35%) at 3 years, and 36% (30–43%) after 3 years (74). Finally, negative symptoms only showed sufficient variability in male subjects, which prevented us from performing a more detailed evaluation of sex differences in symptomatology. It would be interesting to have investigated the interactions between symptom severity and neuroanatomy in females in comparison with males. Further, it is possible that we reached a floor effect, as this population only presents with subthreshold psychotic symptoms.

## Conclusion

In conclusion, sex differences in neuropsychiatric illness and brain anatomy can be a useful tool to better understand disease onset and progression. We present evidence to suggest that males and females at CHR for psychosis present with different patterns of abnormalities in their brain anatomy, their symptomatology, and the relationship between the two. This area of research needs further investigation, to better understand how and why males and females who are placed in the same “high-risk” category may express different disease-related phenotypes. Further investigation of neuroendocrine function but also sex steroids in males and females at CHR for psychosis could be useful in explaining some of the biological underpinnings of the neuroanatomical alterations observed. Furthermore, studies acquiring longitudinal neuroimaging and clinical data on these individuals would give us a better understanding of how sex differences in neuroanatomy might evolve with conversion to psychosis or with remittance of any symptoms. Finally, a better understanding of the sex-specific susceptibility to developing psychosis will help inform more appropriate intervention and treatment measures for male and female CHR individuals to prevent or delay psychosis onset.

## Ethics Statement

This study was carried out in accordance with the recommendations of McGill Institutional Review Board with written informed consent from all subjects. All subjects gave written informed consent in accordance with the Declaration of Helsinki. The protocol was approved by the McGill Institutional Review Board.

## Author Contributions

EG led the data processing and analysis, hypothesis generation, statistical analysis, and interpretation under the supervision of MC and MP. GD provided support for the MRI image processing. MP collected the data under the supervision of AM. JS aided in the interpretation of results. Finally, EG wrote the manuscript under the supervision of MC and MP.

## Conflict of Interest Statement

The authors declare that the research was conducted in the absence of any commercial or financial relationships that could be construed as a potential conflict of interest.

## Footnotes

^1^https://www.mcgill.ca/bic/software/minc.

^2^https://github.com/CobraLab/minc-bpipe-library.

^3^https://github.com/Mouse-Imaging-Centre/RMINC.

## Supplementary Material

Figure S1 Subthreshold sex differences on vertex-wise cortical thickness (CT) measures. Sex-by-diagnosis interactions in CT (*p* < 0.05 unthresholded). Blue denotes areas in which cortex is thinner in females at clinical high risk (CHR) than CTL females, and areas that are thicker in males at CHR compared with CTL males. Pink represent areas in which the opposite is true. Boxplots represent peak voxels in the left and right hemispheres.
