# Supplementary material for: Neuroanatomical and Symptomatic Sex Differences in Individuals at Clinical High Risk for Psychosis
Source: Front Psychiatry. 2017 Dec 22;8:291. doi: 10.3389/fpsyt.2017.00291 (PMC5744013; doi:10.3389/fpsyt.2017.00291)
Supplement: Supplementary file 1 [file Table_1.DOCX]

|  | **CTL Males** | | **CHR Males** | | **CTL Females** | | **CHR Females** | |
| --- | --- | --- | --- | --- | --- | --- | --- | --- |
| **Structure** | **Mean (mm^3^)** | **SD** | **Mean (mm^3^)** | **SD** | **Mean (mm^3^)** | **SD** | **Mean (mm^3^)** | **SD** |
| **Total Brain** | 1,687,625.91 | 88,276.81 | 1,514,650.80 | 1,514,650.80 | 1,434,984.10 | 152,692.74 | 1,289,346.00 | 299,469.54 |
| **Hippocampus, Right** | 2,612.53 | 244.24 | 2,723.46 | 2,723.46 | 2,680.64 | 266.67 | 2,698.69 | 268.38 |
| **Hippocampus, Left** | 2,568.27 | 261.82 | 2,636.62 | 2,636.62 | 2,591.86 | 259.56 | 2,691.77 | 260.93 |
| **Amygdala, Right** | 1,494.07 | 115.05 | 1,513.77 | 1,513.77 | 1,327.93 | 113.41 | 1,309.85 | 134.39 |
| **Amygdala, Left** | 1,481.60 | 110.33 | 1,473.08 | 1,473.08 | 1,303.79 | 125.23 | 1,299.62 | 127.88 |
| **Striatum, Right** | 10,160.80 | 549.91 | 10,013.38 | 10,013.38 | 8,887.93 | 662.09 | 9,066.77 | 772.20 |
| **Striatum, Left** | 10,430.47 | 585.09 | 10,319.85 | 10,319.85 | 9,083.50 | 720.65 | 9,265.54 | 803.60 |
| **Globus Pallidus, Right** | 1,610.67 | 101.97 | 1,563.54 | 1,563.54 | 1,435.50 | 111.55 | 1,508.62 | 155.11 |
| **Globus Pallidus, Left** | 1,778.67 | 125.78 | 1,713.23 | 1,713.23 | 1,547.29 | 129.62 | 1,611.00 | 125.79 |
| **Thalamus, Right** | 7,008.00 | 431.92 | 6,776.92 | 6,776.92 | 5,984.86 | 446.93 | 6,024.31 | 554.49 |
| **Thalamus, Left** | 6,810.20 | 441.43 | 6,632.23 | 6,632.23 | 5,827.36 | 435.17 | 5,905.00 | 532.5 |

**Supplementary Table 1.** Summary of mean volumes with standard deviation (SD) per sex and diagnosis subgroups
