# Supplementary material for: Neuroanatomical and Symptomatic Sex Differences in Individuals at Clinical High Risk for Psychosis
Source: Front Psychiatry. 2017 Dec 22;8:291. doi: 10.3389/fpsyt.2017.00291 (PMC5744013; doi:10.3389/fpsyt.2017.00291)
Supplement: Supplementary file 2 [file Table_2.DOCX]

**Supplementary Table 2.** Summary of the sex by group interaction, sex, and group main effect statistics per structure.

|  | **Sex-by-group interaction** | | **Group Main Effect** | | **Sex Main Effect** | |
| --- | --- | --- | --- | --- | --- | --- |
| **Structure** | **T-value** | **P-value** | **T-value** | **P-value** | **T-value** | **P-value** |
| **Total Brain** | 1.756 | **0.088020** | **-**3.67 | **0.0008** | -5.83 | **<0.0001** |
| **Hippocampus, Right** | -0.732 | 0.4692 | 1.58 | 0.123 | 1.78 | **0.085** |
| **Hippocampus, Left** | -0.128 | 0.8986 | 1.26 | 0.215 | 1.67 | **0.099** |
| **Amygdala, Right** | **-**1.801 | **0.08061** | 3.39 | **0.002** | 2.61 | **0.014** |
| **Amygdala, Left** | -1.495 | 0.14422 | **2.60** | **0.014** | **2.87** | **0.007** |
| **Striatum, Right** | -0.495 | 0.6235 | 2.33 | **0.026** | 1.26 | 0.216 |
| **Striatum, Left** | -0.686 | 0.4973 | 2.43 | **0.020** | 1.19 | 0.243 |
| **Globus Pallidus, Right** | 0.116 | 0.9086 | 1.44 | 0.159 | 1.85 | 0.073 |
| **Globus Pallidus, Left** | 0.271 | 0.788 | 1.29 | 0.206 | 1.43 | 0.163 |
| **Thalamus, Right** | -0.875 | 0.388 | 1.58 | 0.123 | 1.65 | 0.109 |
| **Thalamus, Left** | -0.737 | 0.4660 | 1.70 | **0.099** | 1.46 | 0.153 |
