# Supplementary material for: Neuroanatomical and Symptomatic Sex Differences in Individuals at Clinical High Risk for Psychosis
Source: Front Psychiatry. 2017 Dec 22;8:291. doi: 10.3389/fpsyt.2017.00291 (PMC5744013; doi:10.3389/fpsyt.2017.00291)
Supplement: Supplementary file 3 [file Data_Sheet_1.docx]

**Supplementary materials**

**1. Materials and Methods**

- 1. **Automatic segmentation of basal ganglia volumes:** basal ganglia volumes were extracted from T1 images using the automatic multi-atlas segmentation algorithm, MAGeT Brain, as described in the main text ^1,2^. The bilateral striatum, globus pallidus, thalamus were defined using a three-dimensional reconstruction of serial histological data and were warped to a high-resolution average ^3^, which was then used to label the 21 template scans, and the same procedure as in the main text was performed, generating 21 candidate labels per subject prior to label fusion. Quality control was performed as described in the main text.
  2. **Vertex-wise surface area:** surface-based analyses of basal ganglia structures were performed as described in the main text. The only difference is the use of 1 high-resolution atlas (same as in 1.1), instead of 5 high-resolution atlases.
  3. **Statistical analyses:** we performed an exploratory analysis of the striatum, thalamus, and globus pallidus volumes normalized to total brain volume using the same model described in the main text (sex-by-group interaction with age as a covariate). We corrected for multiple comparisons using a Bonferroni correction for 6 comparisons (setting the threshold for significance at p=0.008).

**2. Results**

2.1. **Basal ganglia volumetric findings:** Striatal volume (as a ratio of TBV) was found to be bilaterally larger in both males and females at CHR relative to controls, however they do not survive the Bonferroni correction for multiple comparisons (p>0.008) (Right: t=2.23, p=0.03, Left: t=2.40, p=0.03). There were no sex differences noted for volumes of the thalamus (right: p=0.39, left: p=0.47) or globus pallidus (right: p=0.91, left: p=0.79). No significant effects were noted for surface-based shape measures. For summary of mean values (± standard deviation (SD)) and statistics see supplementary materials tables 1 and 2.

2.2 **Subthreshold sex differences in surface area of the hippocampus and amygdala**: the sex-by-group interaction did not survive FDR threshold for the hippocampus or amygdala surface areas, so the threshold was set to p<0.05 (uncorrected) to explore subthreshold effects; here we observed an interaction in both the left lateral amygdala surface areas, with males at CHR having enlarged surface area relative to control males, and no difference between controls and CHR females. We found effects in the right amygdala following the same patterns for the males (CHR males larger than control males), and with control females having larger surface area than HR females (Supplementary Figure 2). Subthreshold sex-by-by group interactions were also observed in dorsal hippocampal surface area, with expansions in females at CHR relative to controls, and contractions in males at CHR compared to controls. Exploratory analyses of the striatum, thalamus, and globus pallidus revealed no significant sex-by-group interactions.

**References:**

1. Chakravarty, M. M. *et al.* Performing label-fusion-based segmentation using multiple automatically generated templates. *Hum. Brain Mapp.* **34,** 2635–54 (2013).

2. Pipitone, J. *et al.* Multi-atlas segmentation of the whole hippocampus and subfields using multiple automatically generated templates. *Neuroimage* **101,** 494–512 (2014).

3. Chakravarty, M. M., Bertrand, G., Hodge, C. P., Sadikot, A. F. & Collins, D. L. The creation of a brain atlas for image guided neurosurgery using serial histological data. *Neuroimage* **30,** 359–376 (2006).
