# Supplementary figures and images for: Neuroanatomical and Symptomatic Sex Differences in Individuals at Clinical High Risk for Psychosis
Source: Front Psychiatry. 2017 Dec 22;8:291. doi: 10.3389/fpsyt.2017.00291 (PMC5744013; doi:10.3389/fpsyt.2017.00291)

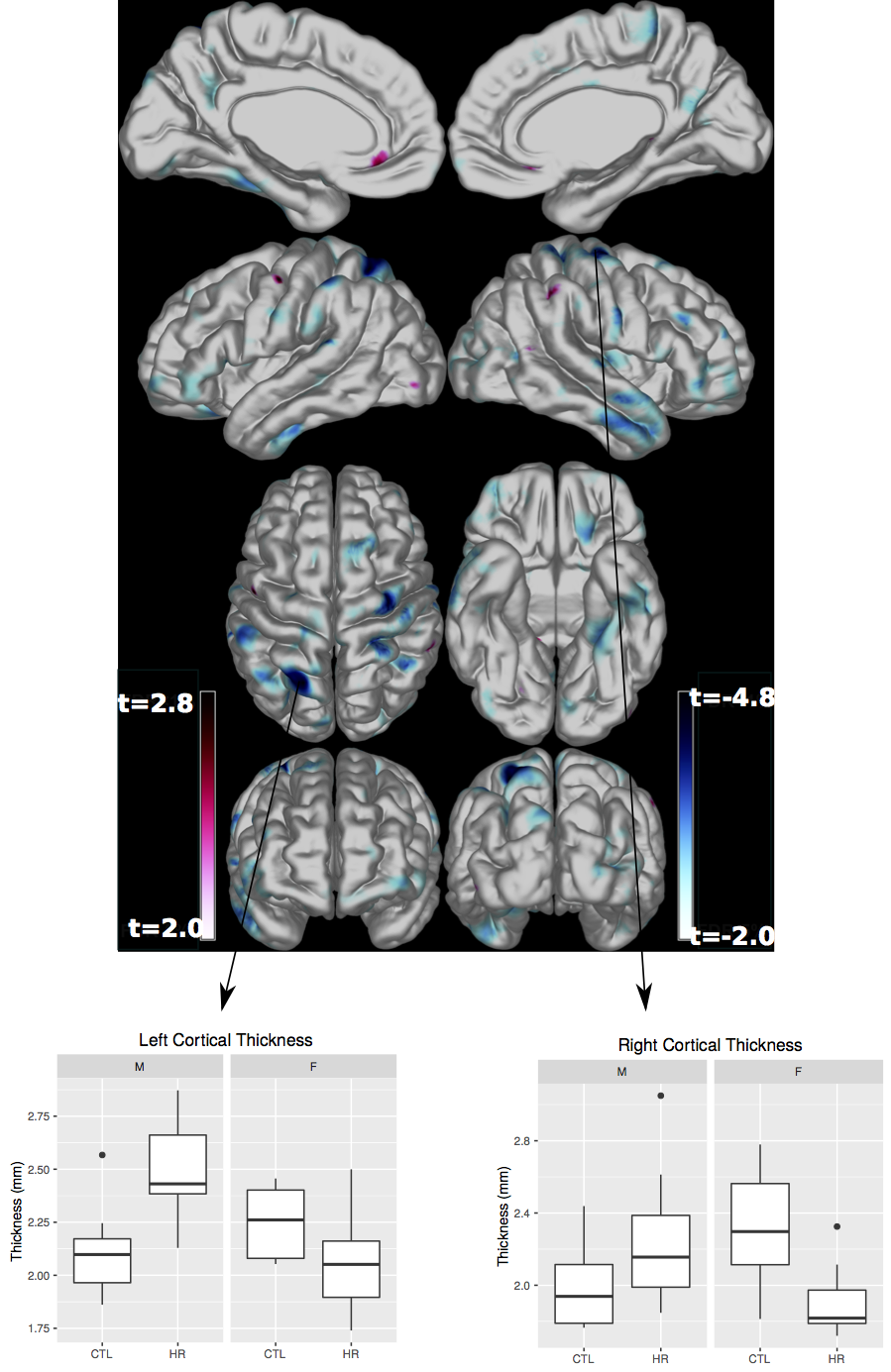

Supplement: Figure S1 — Subthreshold sex differences on vertex-wise cortical thickness (CT) measures. Sex-by-diagnosis interactions in CT (p < 0.05 unthresholded). Blue denotes areas in which cortex is thinner in females at clinical high risk (CHR) than CTL females, and areas that are thicker in males at CHR compared with CTL males. Pink represent areas in which the opposite is true. Boxplots represent peak voxels in the left and right hemispheres. [file Image_1.TIFF]
